# Supplementary material for: Iterative improvement in the automatic modular design of robot swarms
Source: PeerJ Comput Sci. 2020 Dec 7;6:e322. doi: 10.7717/peerj-cs.322 (PMC7924708; doi:10.7717/peerj-cs.322)
Supplement: Supplemental Information 3 [file peerj-cs-06-322-s003.zip › argos3/doc/api/standalone/a00405_source.html]

ARGoS: core/utility/signal\_processing.h Source File


- Main Page
- Related Pages
- Namespaces
- Classes
- Files

- File List
- File Members

# core/utility/signal\_processing.h

Go to the documentation of this file.

```
00001 #ifndef SIGNAL_PROCESSING_H
00002 #define SIGNAL_PROCESSING_H
00003 
00004 #include <argos3/core/utility/math/general.h>
00005 
00006 namespace argos {
00007 
00008    /****************************************/
00009    /****************************************/
00010 
00014    class CStats {
00015 
00016    public:
00017       
00018       CStats();
00019       
00024       inline Real GetMean() const {
00025          return m_fMean;
00026       }
00027       
00034       Real GetVariance() const;
00035 
00042       inline Real GetStdDev() const {
00043          return Sqrt(GetVariance());
00044       }
00045 
00050       void Append(Real f_value);
00051       
00052    private:
00053       
00054       UInt64 m_unCounter;
00055       Real m_fMean;
00056       Real m_fSumOfSquareDiff;
00057    };
00058 
00059    /****************************************/
00060    /****************************************/
00061 
00066    class CRCLowPassFilter {
00067 
00068    public:
00069 
00074       CRCLowPassFilter(Real f_smoothing_factor);
00075 
00081       Real Filter(Real f_input);
00082 
00086       void Reset();
00087 
00088    private:
00089 
00090       Real m_fSmoothingFactor;
00091       Real m_fPreviousOutput;
00092       bool m_bInitialized;
00093    };
00094    
00095    /****************************************/
00096    /****************************************/
00097    
00098 }
00099 
00100 #endif
00101
```

---

Generated on 10 Jul 2018 for ARGoS by 
 1.6.1
